# Computational Studies of Hydroxychloroquine and Chloroquine Metabolites as Possible Candidates for Coronavirus (COVID-19) Treatment

**DOI:** 10.3389/fphar.2020.569665

**Published:** 2020-11-12

**Authors:** Niteen A. Vaidya, Renu Vyas

**Affiliations:** ^1^ ChiroSolve, San Jose, CA, United States; ^2^ MIT School of Bioengineering Sciences & Research, Pune, India

**Keywords:** coronavirus (COVID-19), chloroquine, hydroxychloroquine, drug design, computational toxicity, molecular docking, metabolites, ViridisChem, SARS-CoV-2, reaction mechanism, cheminformatics

## Abstract

The coronavirus disease 2019 or COVID-19 pandemic is claiming many lives, impacting the health and livelihoods of billions of people worldwide and causing global economic havoc. As a novel disease with protean manifestations, it has pushed the scientific community into a frenzy to find a cure. The chloroquine class of compounds, used for decades for their antimalarial activity, have been well characterized. Hydroxychloroquine (HCQ), a less toxic metabolite of chloroquine, is used to treat rheumatic diseases such as systemic lupus erythematosus (SLE), rheumatoid arthritis (RA), juvenile idiopathic arthritis (JIA), and Sjögren’s syndrome. Preliminary studies in non-randomized clinical trials point to the possible use of chloroquine and its derivatives in the treatment of coronavirus. However, more robust clinical studies carried out in the United States, Italy, Australia, and China have shown mixed and inconclusive results and indicate the need for additional research. Cardiac, neurological, and retinal toxicity as well as increasing parasite resistance to these drugs is a major hindrance for their use in a world that is already dealing with antimicrobial resistance (AMR). In this context, we chose to study the monoquinoline analogs of 4-aminoquinoline as well as their metabolites which have the same mechanism of action albeit with lower toxicity. All the compounds were extensively studied computationally using docking, cheminformatics, and toxicity prediction tools. Based on the docking scores against ACE (angiotensin-converting enzyme) receptors and the toxicity data computed by employing the chemical analyzer module by ViridisChem^™^ Inc., the work reveals significant findings that can help in the process of use of these metabolites against coronavirus.

## Introduction

The urgency to find a solution to prevent the catastrophic effects of the COVID-19 pandemic from claiming more lives is driving pharmaceutical companies and governments to study and explore known, commercially available drugs with well-established safety and efficacy profiles to see if they can be repurposed to find effective treatments. Chloroquine, an antimalarial blood schizonticide is being investigated as both treatment and possible prophylaxis against SARS-CoV-2 infection. 2-Hydroxychloroquine (Liu et al., 2020), currently being used for lupus and rheumatoid arthritis, is a less toxic derivative of chloroquine, and is also an antimalarial blood schizonticide with similar clinical indications and side-effects. It is considered effective in inhibiting SARS-CoV-2 infection *in vitro* (Bodor and Buchwald, 2000). Both the drugs share similar chemical structures and mechanisms of acting as a weak base and immunomodulator, but hydroxychloroquine is demonstrated to be 40% less toxic in animals than chloroquine. Both chloroquine (CQ) and 2-hydroxychloroquine (HCQ) have a reputation for being effective and relatively safe treatments in SLE, mild-moderate RA, and Sjögren’s syndrome.

Initial clinical studies have shown some promise in effectiveness of both the drugs, although so far the results have been inconclusive. There is a need for a) more information on their mode of action in relation to the control of these diseases, b) scope for developing formulations that have improved pharmacokinetic and therapeutic properties and safety, and c) further exploring their use in drug combinations not only with other disease modifying agents but also with biologics.

Due to the emergence and spread of chloroquine-resistant strains, other novel drug candidates based on the structure of chloroquine are also being studied. Specifically, two of the 4-aminoquinoline analogs: monoquinoline (MAQ) and bisquinoline (BAQ) (Aguiar et al., 2012) have shown to enhance the activity against chloroquine-resistant parasites. As they possess similar mode of action to that of chloroquine, their effectiveness in inhibiting SARS-CoV-2 infection should be studied as well. Due to our interest in drug design of inhibitors for infectious diseases such as malaria and tuberculosis (Rudrapal and Chetia, 2010; Casagrande et al., 2012; Sahu et al., 2014; Sharma et al., 2014), we decided to explore this hypothesis further using commercial and academic computational tools that are available.

## Materials and Methods

While considering viability of drug candidates, scientists always consider the following factors (ADMET): absorption, distribution, metabolism, elimination, and toxicity.

It is estimated that close to 50% of drug candidates fail because of unacceptable efficacy and that up to 40% of drug candidates have failed in the past due to toxicity. Because of this, in addition to pharmacological properties, ADME/toxicity studies play a crucial role in the success of a drug candidate and therefore occur early in the drug discovery process. While the ADME predictions offer initial toxicological data, detailed acute and chronic health toxicity information is needed for better understanding about the viability of a drug candidate.

In this paper, we provide both the ADME and toxicity studies of our selected candidates to help understand their viability as potential options.

### 4-Amino-7-Chloroquinoline Derivatives as Promising Candidates

4-Amino-7-chloroquinoline (Aguiar et al., 2012; Sahu et al., 2014) is a basic (parent) moiety of chloroquine and hydroxychloroquine and is considered a key intermediate to synthesize the two chloroquines in the lab (*in vitro*). Chloroquine metabolizes to hydroxychloroquine *in vivo*.

On the other hand, 7-chloro-4-hydroxyquinoline (7-chloro-4-quinolone) (Casagrande et al., 2012; Sharma et al., 2014), an antitumor drug is a hydrolysis product (in vivo or in vitro) of 4-amino-7-chloroquinoline. All these four chemicals show significant biological activity and are in clinical use.

**FIGURE 1:**
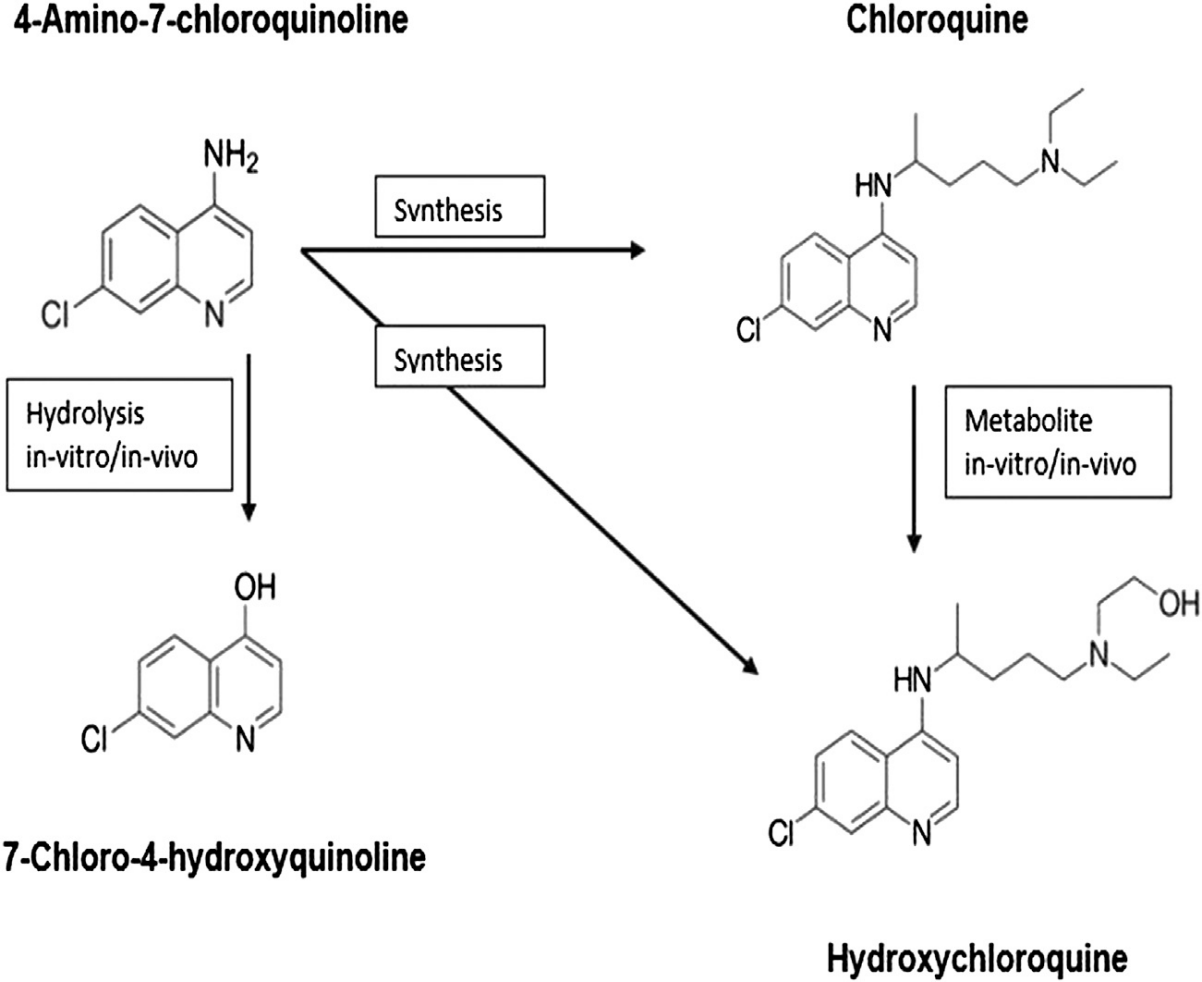
Structure–activity relationship.

The structure–activity relationship studies of 4-aminoquinolines, the parent fragment of chloroquine and hydroxychloroquine, showed that the 7-chloro-4-aminoquinoline nucleus (Figure 1) that is present in pharmacologically active substances displays a broad range of biological activities. Therefore, the incorporation of this active pharmacophore into the structure of new heterocyclic compounds should improve their biological activity.

**FIGURE 2:**
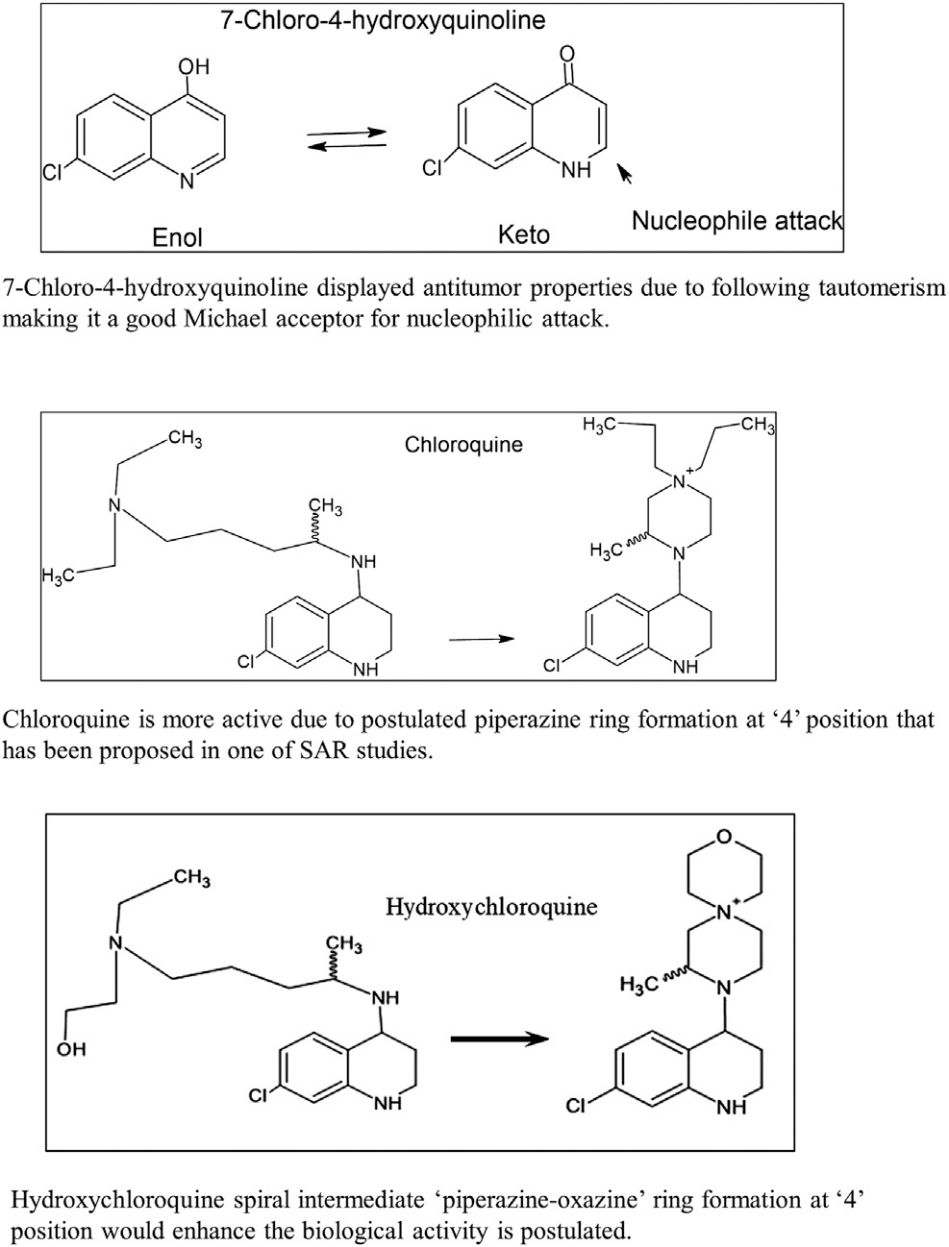
Reaction mechanism for bioactivity.

The first example in Figure 2 shows that 7-chloro-4-hydroxyquinoline (ViridisChem Inc.) displays antitumor properties due to tautomerism, making it a good Michael acceptor for nucleophilic attack. Due to its electrophilic nature (Michael acceptor), the active site of the drug molecule can attract nucleophiles from the host body such as DNA/RNA (deoxyribonucleic acid/ribonucleic acid) as well as other nucleophilic moieties. As an alkylating agent, it preferentially reacts with cancer cells (covalently attaches to its DNA/RNA) to reduce its growth.

The second example in Figure 2 shows that chloroquine’s side chain is a piperazine ring skeleton (Kaur et al., 2010; Guantai et al., 2011). Many drugs have this carbon skeleton where the quinoline ring is substituted at 4-position with the piperazine ring.

The third example in Figure 2 shows that the hydroxychloroquine side chain is coiled around the quinoline ring as piperazine as well as the oxazine ring substituted at 4-position of the parent quinoline ring structure. Many citations in literature show that quinoline is substituted at 4-position with similar heterocyclic moiety.

#### Computational Toxicity Evaluation

Computational toxicity tools are important to reduce time and cost as they obviate the need for animal experiments. We employed commercially available in silico toxicity prediction tool Chemical Analyzer (ViridisChem Inc.), from ViridisChem Inc., to study the four compounds.

ViridisChem has built an extensive toxicity database containing most known chemicals with over 45 different physical, functional, and toxicological properties per chemical. The database was built by collecting and curating experimental data from over 150 sources and by extracting information from US Federal-State and International Regulatory lists. Missing data were estimated by incorporating molecular similarity and fragment properties and by utilizing industry-standard QSAR and other machine-learning prediction models.

The chemical analyzer module offers in-depth ecological, health, and safety-related toxicity scores using internal algorithms based on these properties using OECD (Organization for Economic Co-operation and Development) Guidelines, as well as United Nations and US-OSHA GHS (Globally Harmonized System) Guidelines. Some of the health risk predictions it provides are as follows:

Chronic health: carcinogenicity, genotoxicity, mutagenicity, reprotoxicity, neurotoxicity, and endocrine disruption.
Acute health: skin corrosion/irritation, eye irritation, inhalation toxicity, oral toxicity, and skin sensitization.

Where possible, the toxicological properties including LC50 (species: Daphnia, fish, green algae), LD50 (rat) values are provided to help understand the acute and chronic health impact of the chemicals.

The compounds were extensively studied computationally using docking, cheminformatics, and toxicity prediction tools. The molecular docking was performed and analyzed via the AutoDock Vina docking tool (Morris et al., 2009). The data was analyzed to reveal significant findings that can help in the process of use of these metabolites against coronavirus.

**FIGURE 3:**
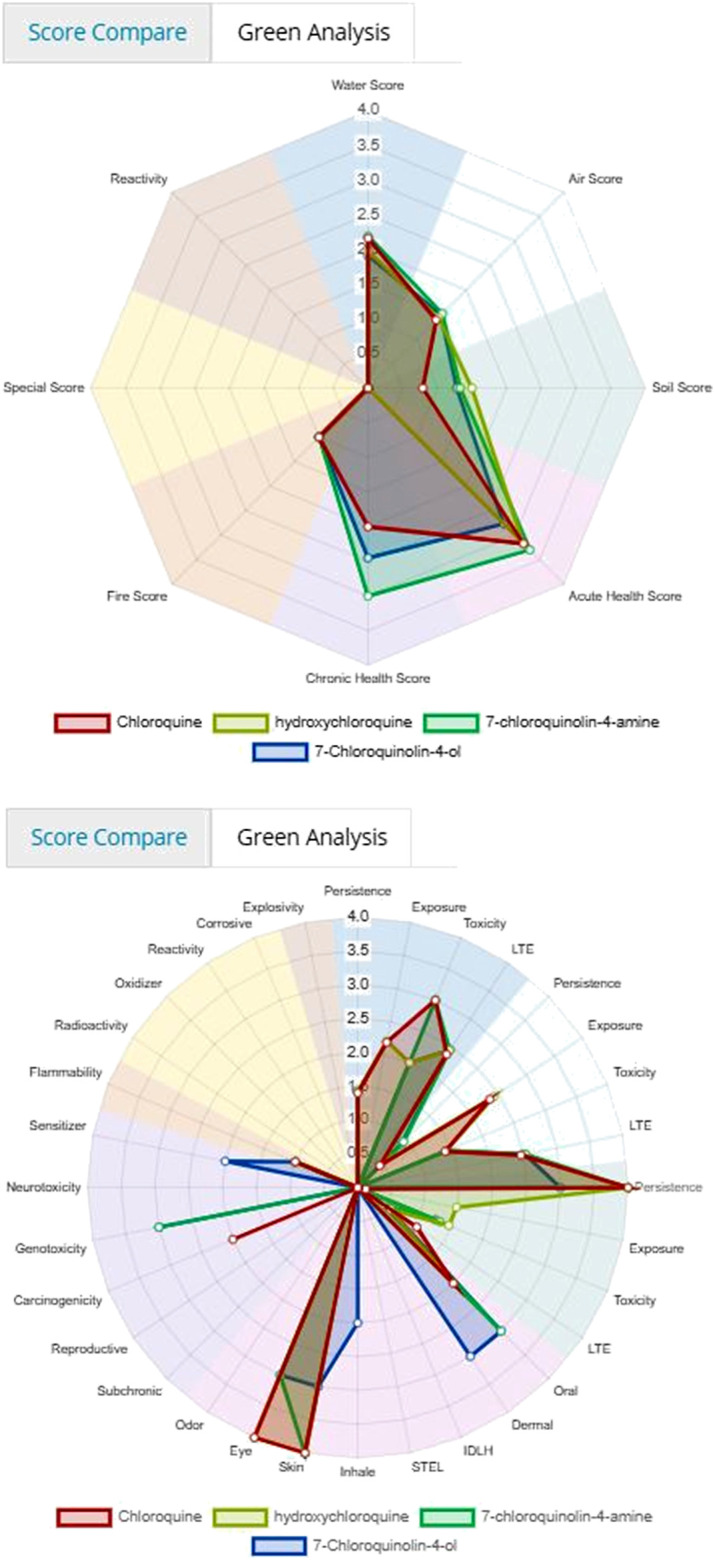
Toxicity score comparison [snapshot of ViridisChem Chemical Analyzer (ViridisChem Inc.)].

**FIGURE 4:**
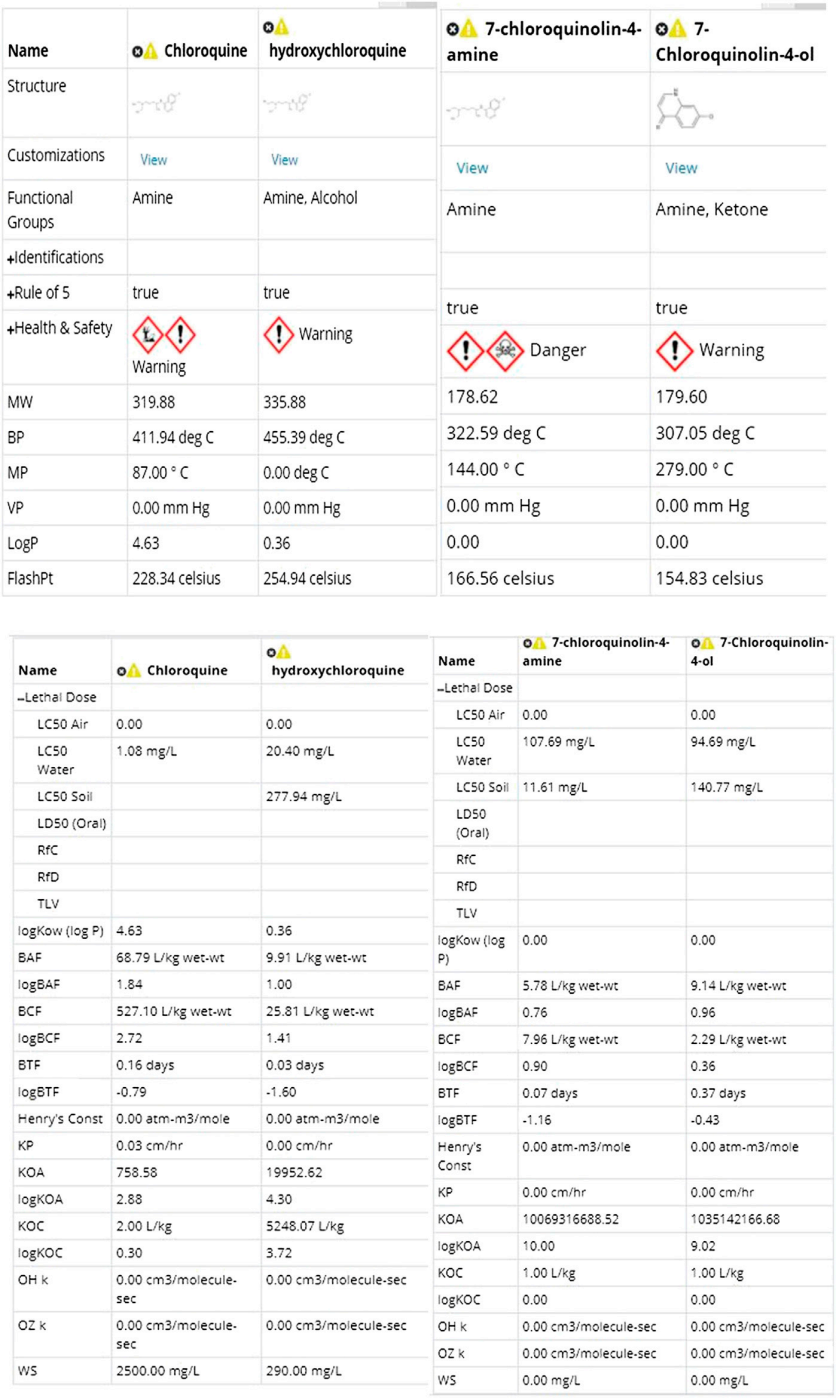
Physical and toxicological properties [snapshot of ViridisChem Chemical Analyzer (ViridisChem Inc.)].

Figure 3 and 4 depicts the comparison among the four molecules processed by the ViridisChem’s module. The area outlined by color-coded lines is indicative of the toxicity of the molecule (larger the area, more toxic the molecule). The scores are calibrated from 0 to 4, where 0 = no (or unknown) toxicity; 1, 2, 3 = increasingly higher toxicity; and 4 = extreme toxicity. Each score was defined using OECD and United Nations Guidelines and utilizes the physical and toxicological property values.

**FIGURE 5:**
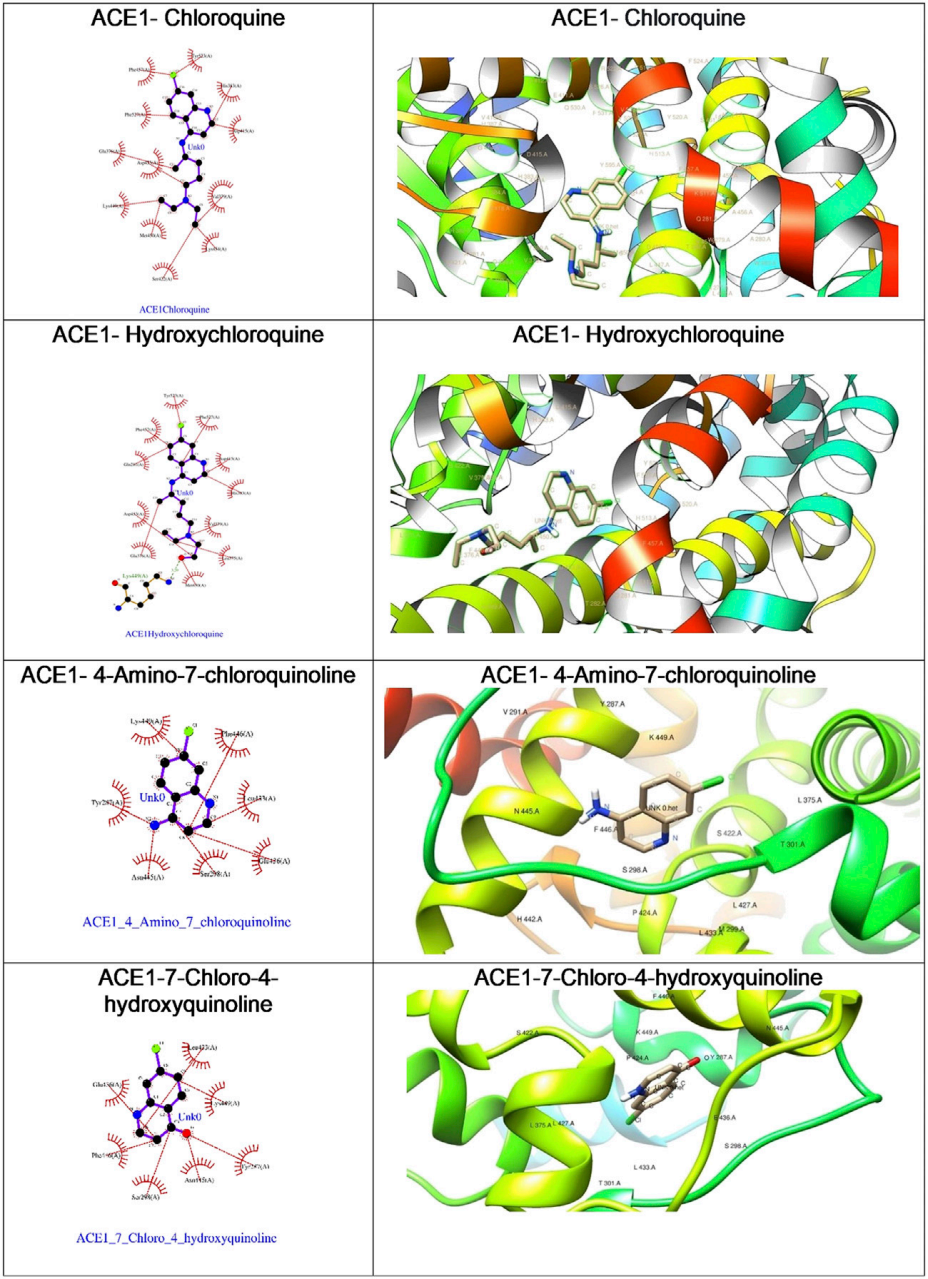
The ligplot diagrams of intermolecular interactions of four compounds in column 1. The second column depicts the best docked pose in the active site of the ACE1 receptor.

**FIGURE 6:**
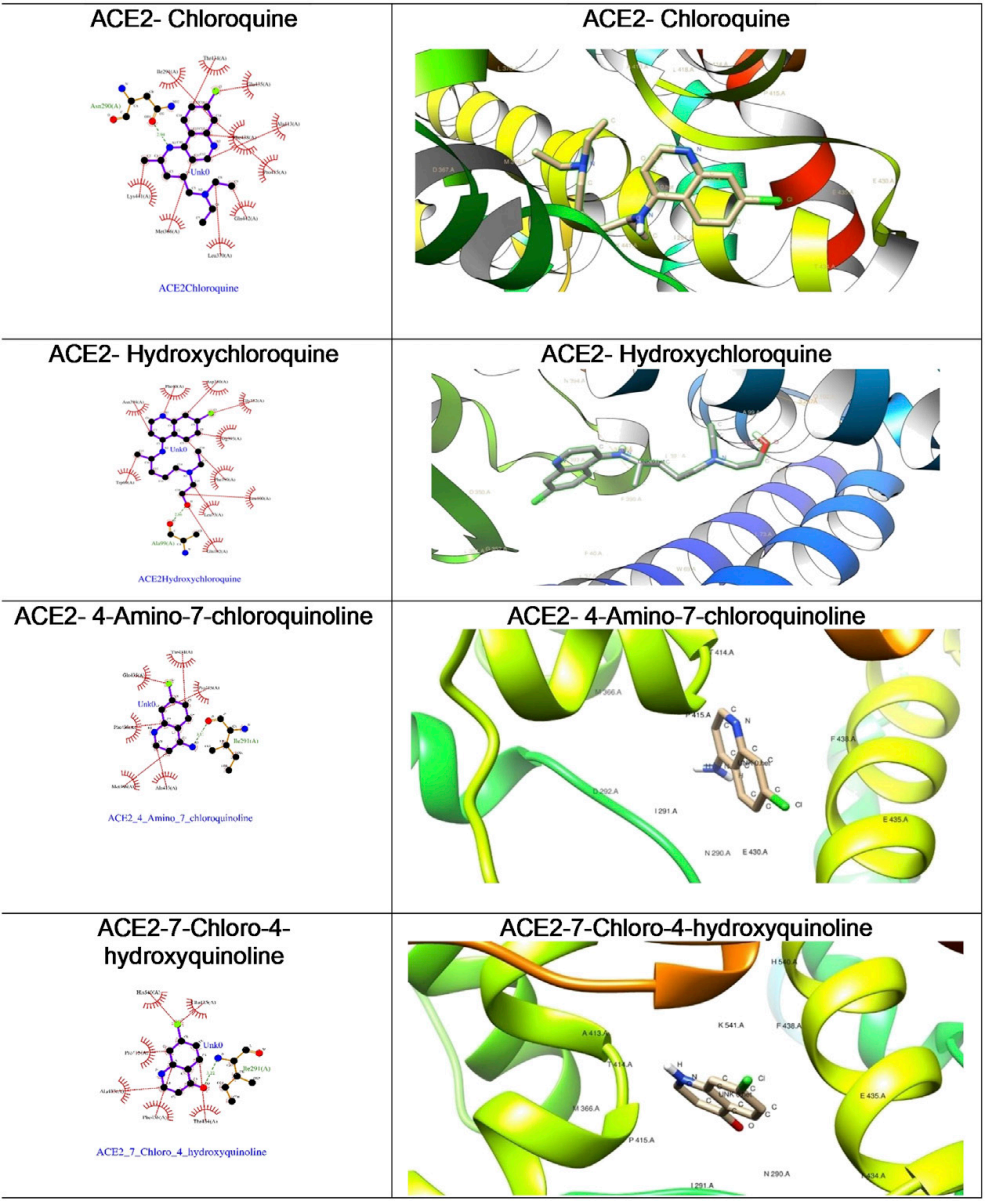
The ligplot diagrams of intermolecular interactions of four compounds in column 1. The second column depicts the best docked pose in the active site of the ACE2 receptor.

As shown in Figure 3 and 4, chloroquine shows higher toxicity (covers a larger area on the spider chart) than the other three molecules. Its high log *K*_o/w_ (log of octanol/water partition coefficient) value and high BCF (bioconcentration factor > 500 L/kg) value also indicate that this compound is persistent and if used in large quantity poses the threat of being a persistent organic pollutant (POP). Table 1 shows some of the health-related toxicity endpoint values that indicate the key differentiation between the compounds. The values serve as early warning of some of the risks and suggest areas of interest for clinical work.

#### Molecular Docking Studies: Molecular Docking of Angiotensin-Converting Enzyme Receptors With Chloroquine and Hydroxychloroquine

##### Preparation of Macromolecule

The protein targets retrieved from RCSB Protein Data Bank were ACE1 (PDB code 6F9T), lactate dehydrogenase (PDB code 1CET), glutathione S-transferases (PDB code 1OKT), and ACE2 (PDB code 6M0J) which served as docking receptors. The proteins were fixed for errors in atomic representations and optimized using AutoDock. The bond orders were assigned to residues; hydrogen atoms were added at pH 7.0. Minimization was carried out using OPLS 2005 force field with a RMSD cut-off value of 0.3 Å.

##### Preparation of Ligands

The 2D structures of the compounds chloroquine, 4-amino-7-chloroquinoline, 7-chloro-4-hydroxyquinoline, and hydroxychloroquine were converted to 3D structures (.pdb) using the ligand preparation AutoDock MGL tool. The tool searches for tautomers and carries out energy minimization by applying the OPLS 2005 force field.

##### Molecular Docking

The molecular docking was performed and analyzed via the AutoDock Vina docking tool (Penna-Coutinho et al., 2011). The receptor grid was centered based on the active site of the protein using receptor grid generation tool. Ligands prepared were flexibly docked in a grid box using AutoDock Vina simulation algorithm. The favorably docked molecules were ranked according to the docking score.

##### Molecular Docking Analysis

With the objective of exploring the binding potential of all the molecules for angiotensin-converting enzyme (ACE) receptors, we performed docking studies as shown in Table 1.

**TABLE 1:** Key health-related toxicity endpoint information.

Toxicity endpoint	Chloroquine	Hydroxychloroquine	4-Amino-7-chloroquinoline	7-Chloro-4-hydroxyquinoline
LC50_water (mg/L)	1.08	20.4	107.69	94.69
LC50_soil (mg/L)	—	277.94	11.61	140.77
Oral_toxicity^a^	2	2	3	3
Dermal toxicity^a^	—	—	—	2
Inhalation toxicity^a^	—	—	—	2
Skin irritation^a^	4	4	4	3
Eye irritation^a^	4	4	3	3
Skin sensitization^a^	—	—	2	2
Genotoxicity^a^	—	—	3	3
Carcinogenicity^a^	2	—	—	—

a Values shown are scores that range from 0 to 4, indicating increasing severity. “—” indicates that the data is not available.

As depicted in Table 2, the docking score of chloroquine is the best indicator of its effectiveness for binding the target. For all four compounds docked against the ACE1 enzyme, lysine 449 is the key amino acid residue involved in binding with the small molecules indicating a similar binding mode of action against the protein. It is now established that the binding to the ACE2 receptor is a critical initial step for SARS-CoV-2 to enter target cells. Interestingly, in case of the ACE2 enzyme, 7-chloro-4-hydroxyquinoline revealed the best docked score hinting at a good binding affinity. The key amino acid residues involved in docking for chloroquine and hydroxychloroquine are different (Asn290 and Ala99, respectively), clearly emphasizing a different binding mode. For the compounds **3** and **4**, the key amino acid residues are identical indicating they must be interacting with ACE2 receptor active site residues in a similar binding mechanism. These compounds can be further explored as potential therapeutic agents for COVID-19.

**TABLE 2:** Molecular docking analysis of chloroquine and hydroxychloroquine with angiotensin-converting enzyme protein. The binding energies were calculated using the AutoDock Vina docking tool (Penna-Coutinho et al., 2011).

Compound	Angiotensin-converting enzyme 1: 6F9T		Angiotensin-converting enzyme 2: 6M0J	
	Amino acids involved in intermolecular interactions	AutoDock score (kcal/mol)	Amino acids involved in intermolecular interactions	AutoDock score (kcal/mol)
Chloroquine	Lys449, Val379	−7.4	Asn290	−6.7
Hydroxychloroquine	Lys449	−6.9	Ala99	−6.2
4-Amino-7-chloroquinoline	Lys449, Glu436, Tyr287, Asn445, Phe443, Phe446, Leu433, Ser298	−6.3	Ile291, Phe438, Glu435, Thr434, Pro415, Ala413	−6.6
7-Chloro-4-hydroxyquinoline	Lys449, Glu436, Tyr287, Asn445, Phe443, Phe446, Leu433, Ser298	−6.4	Ile291, Phe438, Glu435, Thr434, Pro415, Ala413	−6.9

##### Pocket Region of Angiotensin-Converting Enzyme 1 and Angiotensin-Converting Enzyme 2

Angiotensin-1-converting enzyme (ACE) is a monomeric, membrane-bound, zinc- and chloride-dependent peptidyl dipeptidase that catalyzes the conversion of the decapeptide angiotensin I to the octapeptide angiotensin 2 by removing a carboxy-terminal dipeptide. ACE has long been known to be a key part of the renin angiotensin system that regulates blood pressure (Morris et al., 2009). Somatic ACE consists of an intracellular domain, a transmembrane domain and two similar extracellular domains, the amino or N domain, and the carboxyl or C domain. Each of the domains contains a catalytically active site characterized by a consensus zinc-binding motif and a glutamine nearer the carboxyl terminus that also binds zinc. The active site is deep within the central cavity and access by substrates is limited by the cavity’s dimensions (Riordan, 2003).

ACE1 and ACE2 proteins, according to the most recent research, are vasoactive enzymes involved in the pathogenesis of COVID-19 (Clarke and Turner, 2012). In this study a structure of angiotensin-1-converting enzyme (ACE1) with a PDB ID 6F9T and ACE2 having PDB ID 6M0J have been employed. ACE1 structure consists of both N and C domains. HEXXH domain is present in both domains. The catalytic zinc ion is at the center of a highly coordinated system involving His383, His387, and Glu411 of cACE (His361, His365, and Glu389 in nACE) and a potential bidentate interaction with the zinc‐binding carboxylate group of the inhibitors (Rabi et al., 2020). ACE2 contains a single zinc-binding catalytic domain, which is a carboxypeptidase with preference for carboxy-terminal hydrophobic or basic residues and is not affected by ACE inhibitors (Cozier et al., 2018).

Coronaviruses use the homotrimeric spike glycoprotein (Song et al., 2018; SwissADME, 2012; UNECE) (comprising an S1 subunit and S2 subunit in each spike monomer) on the envelope to bind to their cellular receptors. Such binding triggers a cascade of events that leads to the fusion between cell and viral membranes for cell entry (Lan et al., 2020). Previous cryo-electron microscopy studies of the SARS-CoV spike protein and its interaction with the cell receptor ACE have shown that receptor binding induces the dissociation of the S1 with ACE2, prompting the S2 to transit from a metastable pre-fusion to a more-stable post-fusion state that is essential for membrane fusion 9–12 (Bosch et al., 2003). Therefore, binding to the ACE2 receptor is a critical initial step for SARS-CoV to enter target cells.

We were curious to understand and compare the binding mechanism of chloroquine and hydroxychloroquine in both malaria and COVID-19 implicated target proteins. We performed docking simulations under similar conditions as shown in Figure 5 and Figure 6. The details are discussed in the section below.

##### Docking of Chloroquine and Hydroxychloroquine to Malaria Target Proteins

**FIGURE 7:**
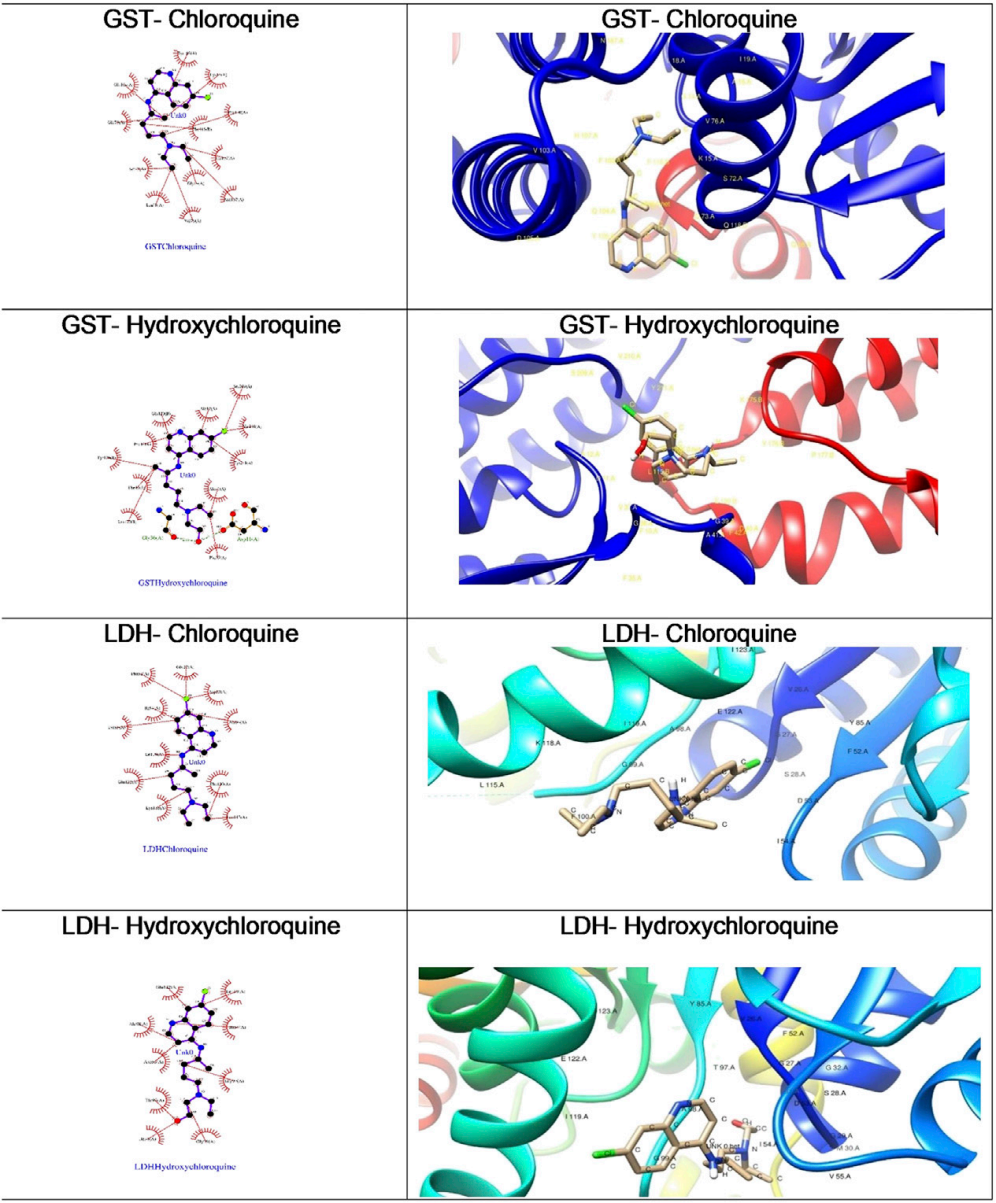
Ligand interaction diagrams and best docked poses of CQ and HCQ against malaria target proteins GST and LDH. GST, glutathione S-transferase; LDH, lactate dehydrogenase.

The *Plasmodium falciparum* lactate dehydrogenase enzyme (PfLDH) (Guantai et al., 2011) has been considered as a potential molecular target for antimalarials due to this parasite’s dependence on glycolysis for energy production. Because the LDH enzymes found in *P. vivax*, *P. malariae*, and *P. ovale* (pLDH) all exhibit ∼90% similarity to PfLDH, it would be desirable to have new anti-pLDH drugs, particularly ones that are effective against *P. falciparum*, the most virulent species of human malaria. Glutathione S-transferases (GSTs), on the other hand, occur abundantly in most organisms, and catalyze the intracellular detoxification of numerous substances (including chemotherapeutic agents), thus playing a major role in the development of drug resistance. Both these proteins are considered as potential targets for malaria. Docking of chloroquine and hydroxychloroquine to malarial targets suggested that chloroquine showed better binding affinity to the malarial protein (Table 3). This similar case was found in binding of chloroquine to angiotensin-converting enzyme 1. When the ligand interaction diagram was compared for both it was observed that the ligand binding sites for both the proteins is the same. Thus, it could be said that there is no significant change in the ligand conformation when docked to either malarial or COVID-19 potential target proteins. The ligand interaction diagrams and docked poses are further highlighted in Figure 7.

**TABLE 3:** Molecular docking analysis of chloroquine and hydroxychloroquine with lactate dehydrogenase and glutathione S-transferase proteins. The binding energies were calculated using the AutoDock Vina.

Compound	Lactate dehydrogenase: 1CET		Glutathione S-transferase: 1OKT	
	Amino acids involved in intermolecular interactions	AutoDock score (kcal/mol)	Amino acids involved in intermolecular interactions	AutoDock score (kcal/mol)
Chloroquine	Glu122, Ala98, Asp53, Ile119, Ile54	−6.2	Tyr108, Phe116	−6.8
Hydroxychloroquine	Glu122, Ala98, Asp53, Ile119, Ile54	−5.7	Asp11, Gly36	−5.8

**TABLE 4:** Molecular docking analysis with angiotensin-converting enzyme protein.

Compound	Angiotensin-converting enzyme 1: 6F9T		Angiotensin-converting enzyme 2: 6M0J	
	Amino acids involved in intermolecular interactions	AutoDock score (kcal/mol)	Amino acids involved in intermolecular interactions	AutoDock score (kcal/mol)
Desethylchloroquine	Val379, Asp453, Phe527, Glu376, Thr282, Gln281, Asn277, Trp279, Tyr523, Phe457, His383, Asp415	−6.6	Arg393, Leu391, Leu73, Phe390, Phe40, Asp350, Asn394	−6.0
Bisdesethylchloroquine	Val379, Glu376, Asp453, Thr282, Asp415, Tyr253, Phe457, His383, Phe527	−7.0	Ala99, Arg393, Phe390, Leu391, Asn394, Trp69, Phe40, Asp350, Gly352	−6.1

Both chloroquine and hydroxychloroquine molecules have exhibited more preference toward the glutathione S-transferase target as against lactate dehydrogenase as evident from the higher binding affinities.

### Chloroquine Metabolites

One of the goals of metabolite characterization is to identify the metabolic pathways and to determine whether any potentially reactive or less toxic metabolites (soft drugs) (Bodor and Buchwald, 2000) are formed. It is generally accepted that toxicities can stem from drug bioactivation *in vivo*. The metabolite characterization of a new chemical entity (NCE) in various drug discovery stages is crucial in assessment of the safety of a drug for human use. The identification of metabolites (Aguiar et al., 2012) may reveal the metabolically labile portions of a molecule in a particular drug series. This information allows synthetic chemists to synthesize compounds that are less susceptible to metabolism and, consequently, have a lower elimination rate and a longer half-life.

Metabolism of most drugs is mediated by the cytochrome P450 system. These kinds of compounds are ideal for producing specific action at the site of application without affecting the rest of the body. There are two major pathways for metabolism:

1. Phase I: biotransformation reactions catalyzed by enzymes (i.e., cytochrome P450), including oxidation, reduction, and hydrolysis. For example, oxidation of aliphatic or aromatic carbon and N-oxidation.
2. Phase II: biotransformation reactions that involve addition of bulky and polar groups through conjugation to a nucleophilic site on the drug molecule. For example, glucuronidation and sulfation.

Both phase I and phase II metabolisms may occur in parallel for particular compounds.

Metabolism of chloroquine into “2-hydroxychloroquine or hydroxychloroquine” and “4-amino chloroquine” is a phase I pathway. The hydroxylation or oxidation of heterocyclic atoms (i.e., nitrogen) is a common phase I oxidative reaction. The metabolites generated by the oxidation at the N-atom are known as N-oxides.

Chloroquine and hydroxychloroquine have some common metabolites such as desethylchloroquine and bisdesethylchloroquine.

#### Computational Toxicity Analysis Using ViridisChem Chemical Analyzer

**FIGURE 8:**
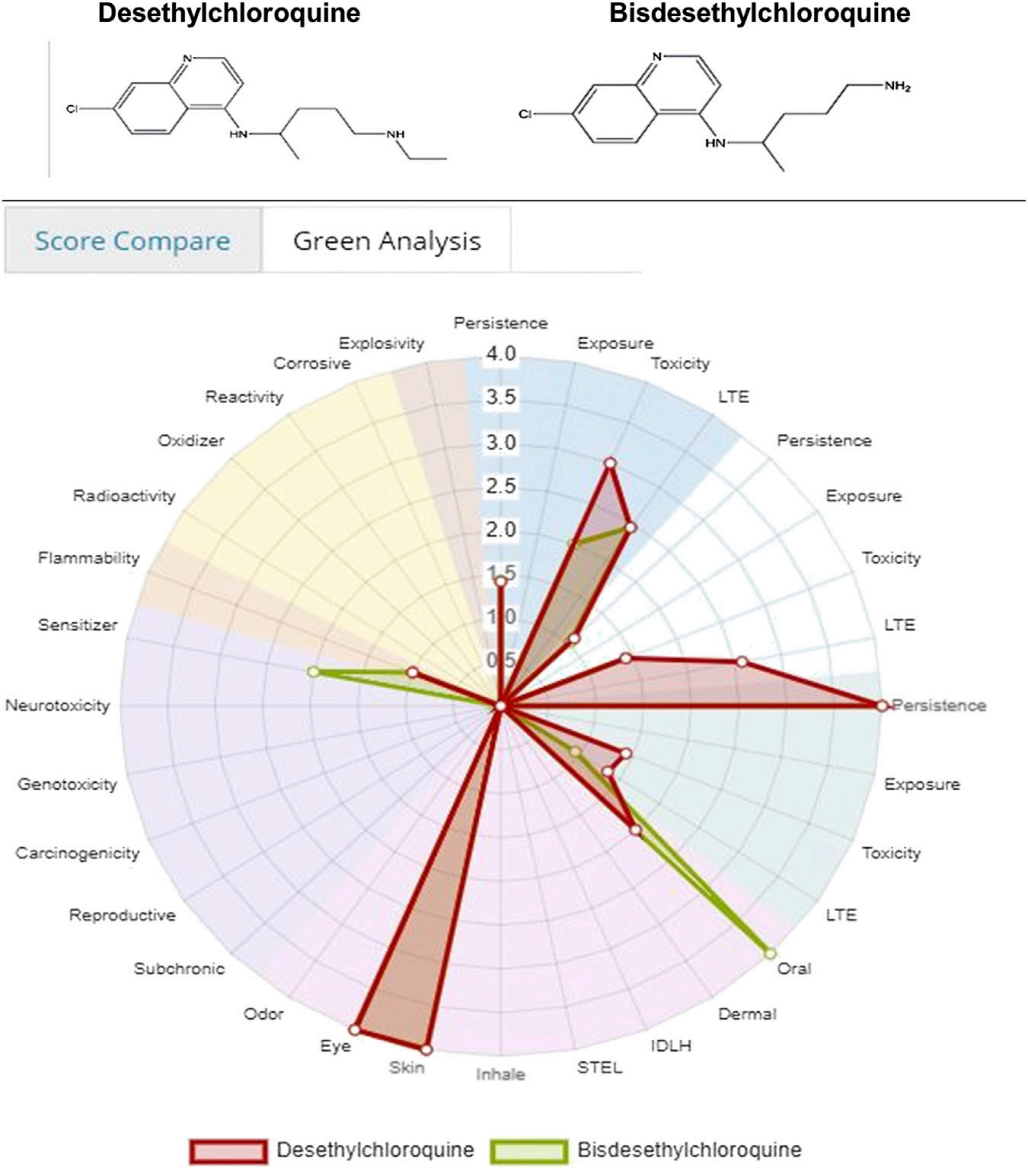
Toxicity score comparison between two metabolites [snapshot of ViridisChem Chemical Analyzer (ViridisChem Inc.)].

**FIGURE 9:**
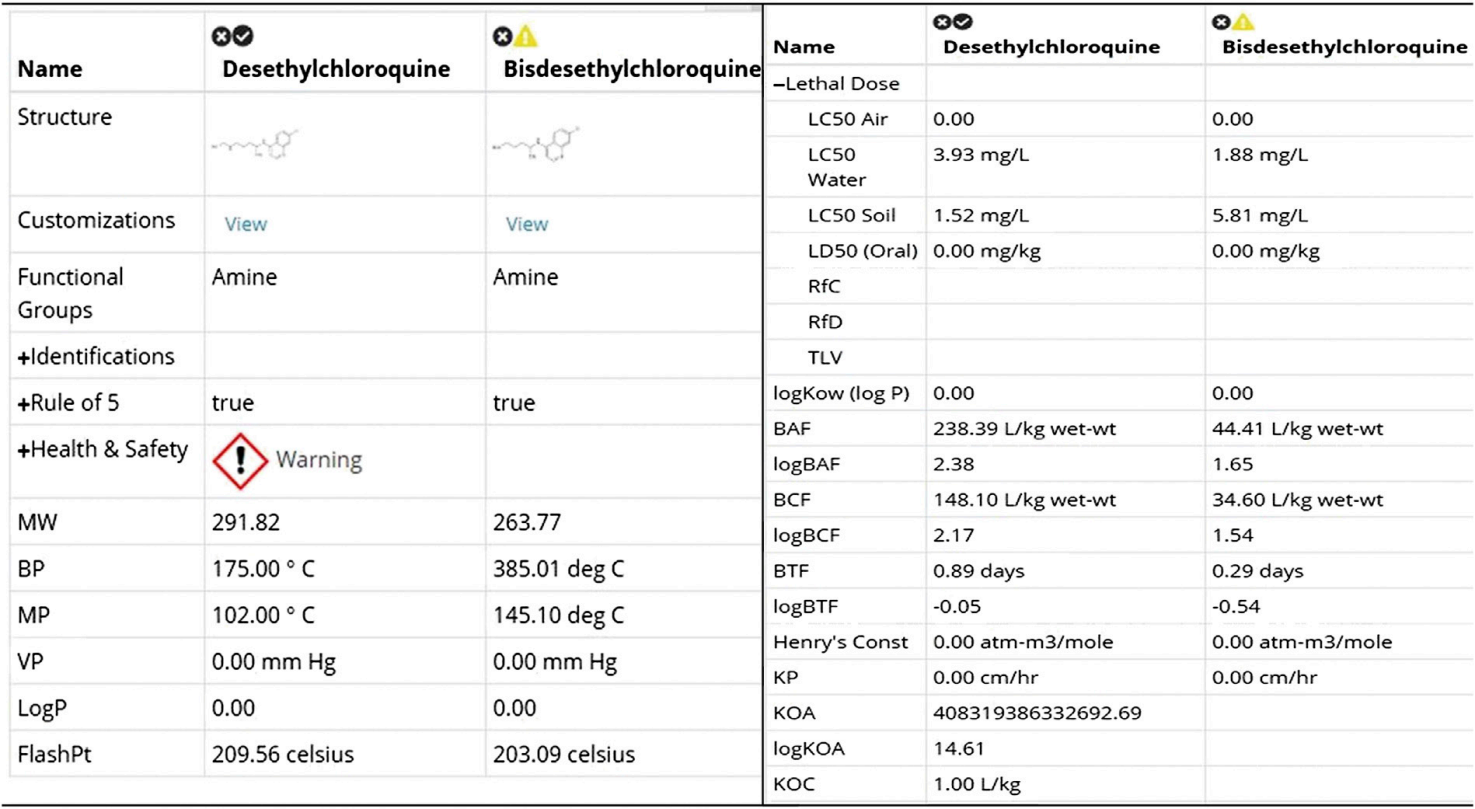
Physical and toxicological properties of the two metabolites [snapshot of ViridisChem Chemical Analyzer (ViridisChem Inc.)].

Both Figures 8, 9 indicate that among the two metabolites desethylchloroquine and bisdesethylchloroquine, the former has higher acute toxicity. Both metabolites are also highly toxic in water and soil. If either of these molecules are pursued, product development should address these issues. Neither of these molecules raise any alarms in terms of health-related toxicity. Therefore, we recommend that further studies should be pursued to assess their viability in the treatment of COVID-19.

#### Molecular Docking Analysis Using AutoDock Vina Docking Tool

We further probed the *in silico* mechanism of action of the metabolites against the COVID-19 receptors ACE1 and ACE2 using our standard docking protocol.

**FIGURE 10:**
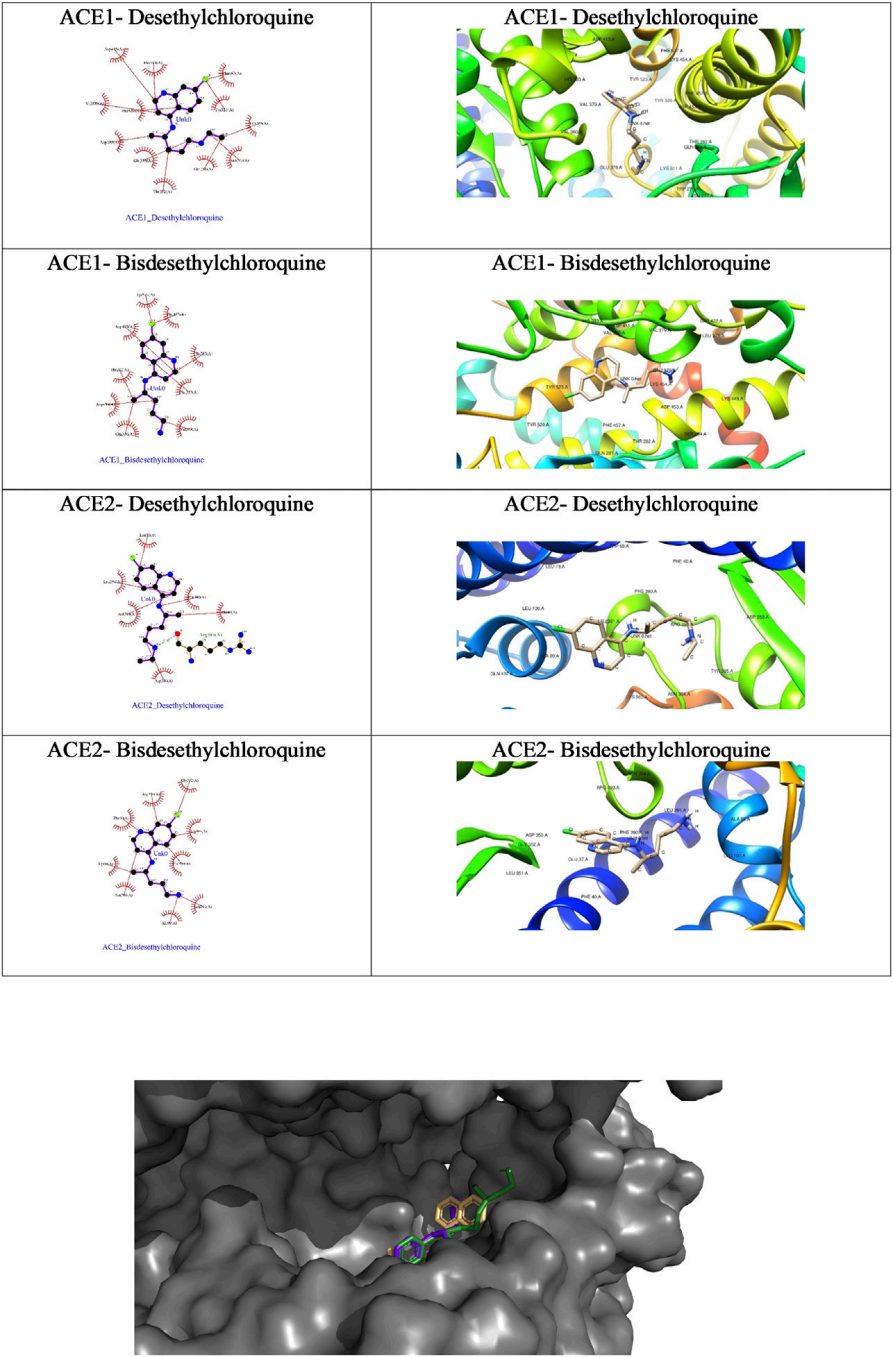
Ligplot view and docked poses of the CQ and HCQ metabolites with ACE1 and ACE2.

As is evident from the tabulated docking results shown in Table 4, both the metabolites showed better binding affinity with ACE1 compared to ACE2, though bisdesethylchloroquine yielded superior results against both the receptors. As we can see in the surface view diagram in Figure 10, the pi ring system of the docked conformation of the bisdesethyl derivative fits snugly into the active site leading to better binding affinity. In the case of desethyl, the longer aliphatic chain might be preventing a good fit of the conformation. The residues in the binding pocket are the same indicating an identical mode of binding of the metabolites with both the enzymes implicated in COVID-19. Therefore, we suggest that further investigations for assessing their potential as therapeutic agents against COVID will be essential.

### Cheminformatics Studies

Further, we studied the cheminformatics properties of all the above compounds to understand their similarities and drug-likeness and the results are tabulated in Table 5. All the compounds have similar properties in terms of bioavailability and obey the Lipinski rule of five.

**TABLE 5:** Cheminformatics data for compounds chosen in this study. The physio-chemical descriptors were computed using the SwissADME server (ViridisChem Inc.).

**Compounds**	**Molecular weight**	**Num. rotatable bonds**	**Log P**_**o/w**_	**Lipinski**	**Drug-likeness**	**Lead-likeness**	**Gi absorption**	**BBB permeant**	**Bioavailability score**
Chloroquine	319.87 g/mol	8	4.63	Yes	Yes	No	High	Yes	0.55
Hydroxychloroquine	335.87 g/mol	9	3.58	Yes	Yes	No	High	Yes	0.55
4-Amino-7-chloroquinoline	178.62 g/mol	0	2.26	Yes	Yes	No	High	Yes	0.55
7-Chloro-4-hydroxyquinoline	179.60 g/mol	0	1.21	Yes	Yes	No	High	Yes	0.55
Desethylchloroquine	291.82	7	3.43	Yes	Yes	No	High	Yes	0.55
Bisdesethylchloroquine	263.77	5	2.64	Yes	Yes	No	High	Yes	0.55

Since the metabolites show a similar cheminformatics profile as the known parent compounds chloroquine and hydroxychloroquine, we conclude that they can be taken forward for potential investigation by research groups for further development as COVID-19 antagonists. Furthermore, the bisdesethylchloroquine compounds with lower rotatable bonds are predicted to have better oral bioavailability, which could prove to be pharmacologically important.

## Conclusion

We have carried out an in-depth computational study of the current drugs chloroquine and hydroxychloroquine and their precursors and metabolites. Based on molecular docking analysis, computation toxicity studies, and the cheminformatics properties, we observe that the two metabolites desethylchloroquine and bisdesethylchloroquine have similar binding modes of action and drug-like properties albeit with significantly lower toxicity values compared to the parent CQ and HCQ molecules. The bisdesethylchloroquine has demonstrated a relatively better profile in terms of target binding, lower computational toxicity, and better cheminformatics profile. Hence, they can be further explored in the coronavirus clinical trials to ascertain their use in anti-viral therapy for combating this pandemic. We hope these computational studies will drive development of relevant assays for their establishment as potential COVID-19 antagonists.

## Data Availability Statement

All datasets presented in this study are included in the article/supplementary material.

## Author Contributions

NV, CTO, ChiroSolve, is an industry expert in Chiral Resolution with over 30 years of drug-development experience. He provided the detailed information about the analogs and metabolites; their toxicity evaluation; and why they should be considered as possible candidates to treat COVID-19. RV, a known molecular modeling expert with extensive experience in cheminformatics, organic chemistry, protein modeling, and drug design provided the comprehensive analysis using docking, cheminformatics, and toxicity prediction tools to help support the recommendations provided by NV.

## Conflict of Interest

NV was employed by the company PointCare Systems, Inc. (DBA ChiroSolve) based in San Jose, CA. He also serves as a technical adviser to the company ViridisChem Inc.

The remaining authors declare that the research was conducted in the absence of any commercial or financial relationships that could be construed as a potential conflict of interest.
